# Psychometric evaluation, using Rasch analysis, of the WHOQOL-BREF in heroin-dependent people undergoing methadone maintenance treatment: further item validation

**DOI:** 10.1186/s12955-014-0148-6

**Published:** 2014-10-03

**Authors:** Kun-Chia Chang, Jung-Der Wang, Hsin-Pei Tang, Ching-Ming Cheng, Chung-Ying Lin

**Affiliations:** Department of General Psychiatry, Jianan Psychiatric Center, Ministry of Health and Welfare, Tainan, Taiwan; Department of Public Health, College of Medicine, National Cheng Kung University, 1 University Road, Tainan, 70101 Taiwan; Departments of Internal Medicine and Occupational and Environmental Medicine, National Cheng Kung University Hospital, Tainan, Taiwan; Department of Psychiatry, Tainan Municipal An-Nan Hospital-China Medical University, Tainan, Taiwan

**Keywords:** Psychometrics, Quality of life, Rasch model, Substance abuse

## Abstract

**Background:**

The brief version of World Health Organization Quality of Life assessment (WHOQOL-BREF), a useful outcome measure for clinical decision making, has been evaluated using classical test theory (CTT) for psychometric properties on heroin-dependent patients. However, CTT has a major disadvantage of invalid summated score, and using Rasch models can overcome the shortcoming. The purpose of this study was using Rasch models to evaluate the psychometric properties of the WHOQOL-BREF for heroin-dependent patients, and the hypothesis was that each WHOQOL-BREF domain is unidimensional.

**Methods:**

Two hundred thirty six participants (24 females, mean [SD] age = 38.07 [7.44] years, first used heroin age = 26.13 [6.32] years), with a diagnosis of opioid dependence, were recruited from a methadone maintenance treatment program. Each participant filled out the WHOQOL-BREF. Parallel analysis (PA) and Rasch rating scale models were used for statistical analyses.

**Results:**

Based on the PA analyses, four domains of the WHOQOL-BREF were unidimensional. The Rasch analyses showed three negatively worded items (2 in Physical and 1 in Psychological) reported as misfits that may not contribute to the Physical and Psychological domains; one positively worded item in the Physical domain may be redundant. All values for the separation indices were above 2 except for the person separation index in the Physical domain (1.93). Category functioning and item independency of four WHOQOL-BREF domains were supported by the Rasch analyses, and there were 5 items showing the differential item function (DIF) for positive versus negative HIV (human immunodeficiency virus) infection.

**Conclusions:**

The WHOQOL-BREF is a valid outcome measure for assessing general quality of life for substance abusers in terms of physical, psychological, social, and environmental factors. It can also be used as a treatment outcome measure to evaluate the effect of treatments for substance abusers. However, the three misfit negatively worded items should be used with caution because the substance abuser may not fully understand their meaning. Future research may apply cognitive interviews to determine the cognitive functioning of substance abusers and their interpretation of negatively worded items.

## Background

Substance abuse, such as heroin addiction, may cause economic burdens for individual users and for the society as a whole [1,2]. Substance abusers are likely to have comorbid infectious diseases, for example, the human immunodeficiency virus (HIV), hepatitis B virus (HBV), and hepatitis C virus (HCV), because of needle sharing [3]. Therefore, substance abusers may have physically and mentally multiple negative consequences as well as those applicable to their social relationships [4], and they are reported to have a poorer quality of life (QoL) than those who are not substance-dependent [5].

QoL is used as an important outcome measure for healthcare decision-making and for evaluating intervention effects [6,7], such as the effect of medicine [8], and studies e.g. [9,10] have used QoL to examine the effect of maintenance treatment on substance abusers. Of the QoL instruments commonly used in maintenance treatment studies, the brief version of the World Health Organization Quality of Life assessment (WHOQOL-BREF) has been suggested to be the most suitable for assessing the global QoL in research on addiction behavior [6,11]. The WHOQOL-BREF has been translated into various languages, including the traditional Chinese used in Taiwan, and the Taiwan version of WHOQOL-BREF has been suggested as valid and reliable (α = 0.70-0.77, comparative fit index [CFI] = 0.89) [12]. Many studies have established the psychometric properties of the WHOQOL-BREF in different populations (e.g., community-dwelling older people [13], people with schizophrenia [14], and depressed people [15]); however, almost no studies have examined substance abusers. To the best of our knowledge, only one recent study [6] has used classical theory test (CTT) methods to evaluate the psychometric properties of the WHOQOL-BREF as applied to substance abusers.

However, using only CTT methods is insufficient for clinicians to understand the psychometric properties of the WHOQOL-BREF. Specifically, CTT methods treat raw scores and item responses to rating scales as interval data, and this may yield invalid scores. Therefore, there is a trend toward using Rasch analysis, a modern statistical method that can transform the ordinal scores of polytomous items into interval scores for the purpose of psychometric evaluation [16]. Although Rasch models have the main weakness of being a complicated model theory in terms of mathematical equations that are hard for clinicians to understand [17], it has the following strengths: (1) the validity of the items can be individually analyzed to determine any redundancy, which may not be detected by CTT; (2) item difficulty can be estimated; (3) an ordinal-to-interval conversion table can be produced that can help clinicians use the items to understand the latent traits of respondents [18-20]. In addition, a number of ordered polytomous Rasch models, such as the partial credit model (PCM) and the rating scale model (RSM), have been used in QoL instruments that are rated on a Likert scale [18].

Previous psychometric evaluations for the WHOQOL-BREF on substance abusers have used mainly CTT methods. Because Rasch models can detect items that are out-of-concept or redundant and can precisely measure the latent QoL of a heroin user using an ordinal-to-interval conversion table, the purpose of this study was to use several Rasch models to examine the psychometric properties of the WHOQOL-BREF in a heroin-dependent sample in Taiwan.

## Methods

### Participants and procedures

The Hospital Ethics Committee of Jianan Psychiatric Center approved this study (IRB number: JMH9601).

All participants (*n* = 236) were recruited from a methadone maintenance treatment (MMT) program and, based on the *Diagnostic and Statistical Manual of Mental Disorders, 4th edition* (DSM-IV) criteria, were diagnosed with opioid dependence by qualified psychiatrists from the Jianan Psychiatric Center. After they had expressed willingness to participate, each participant filled out and signed an informed consent; completed a structured questionnaire, including demographic data, information about substance use, and the WHOQOL-BREF, and then they underwent a series of laboratory tests, including HIV, HBV, and HCV tests.

### Instrument—The WHOQOL-BREF

The WHOQOL-BREF Taiwan version contains 28 items with 26 standard items from the original WHOQOL-BREF and 2 Taiwanese national items [12]. Of the 28 items, 2 are generic items that test overall QoL and general health (i.e., “How would you rate your quality of life?” and “How satisfied are you with your health?”); the remaining 26 are in the Physical (7 items), Psychological (6 items), Social (4 items), and Environment (9 items) domains. All items are scored from 1 to 5, and 3 items (Ph1, Ph2, and Ps6; Table 1) are reversely coded. The 2 Taiwanese national items are, respectively, in the Social (S4) and Environment (E9) domains. Domain score calculation has been reported elsewhere [13], and potential scores for each domain ranged from 4 to 20, with a higher score representing a better QoL. In addition, satisfactory psychometric properties have been established for the WHOQOL-BREF Taiwan version [12].

**Table 1:** **Item difficulty, Rasch fit statistics for each item (** ***N*** **= 236)**

**Domain**	**Mean (SD)**	**Difficulty**	**Infit**	**Outfit**
**Item**				
**Physical**	12.18 (1.92)	--	--	--
*Ph1: Pain and discomfort*	2.57 (1.00)	−0.11	**1.77**	**1.89**
*Ph2: Medication*	2.62 (1.00)	−0.81	**1.56**	**1.66**
Ph3: Energy and fatigue	3.18 (0.87)	0.42	0.67	0.67
Ph4: Mobility	3.62 (0.84)	−0.53	0.88	0.81
Ph5: Sleep and rest	2.99 (1.00)	0.80	0.93	0.95
Ph6: Activities of daily living	3.31 (0.79)	0.15	**0.47**	**0.46**
Ph7: Work capacity^a^	3.35 (0.94)	0.08	0.77	0.73
**Psychological**	12.33 (2.47)	--	--	--
Ps1: Positive feelings	2.43 (1.01)	1.50	1.16	1.17
Ps2: Spirit/ religion/ beliefs	3.16 (1.12)	−0.04	0.92	0.82
Ps3: Think	3.16 (1.00)	−0.04	0.71	0.69
Ps4: Body image	3.54 (0.94)	−0.91	1.08	1.04
Ps5: Self-esteem	3.36 (0.89)	0.78	1.01	0.98
*Ps6: Negative feelings* ^*a*^	2.84 (0.97)	−0.03	**1.56**	**1.63**
**Social**	12.98 (3.00)	--	--	--
S1: Personal relationship	3.39 (0.81)	−0.51	0.87	0.89
S2: Sexual activity	3.03 (1.02)	0.72	1.01	0.98
S3: Social support	3.38 (0.94)	−0.48	1.20	1.11
S4: Being respected^a^	3.17 (0.91)	0.27	0.81	0.76
**Environment**	12.89 (2.65)	--	--	--
E1: Safety and security	3.25 (1.01)	−0.02	1.08	0.99
E2: Physical environment	3.24 (0.94)	0.01	1.05	1.05
E3: Financial resources	2.43 (1.06)	1.76	0.99	0.97
E4: Information acquiring	3.01 (0.98)	0.51	0.84	0.83
E5: Leisure activities	3.00 (1.01)	0.55	1.05	1.06
E6: Home environment	3.55 (0.79)	−0.76	0.77	0.73
E7: Health services	3.53 (0.85)	−0.72	1.14	1.16
E8: Transportation	3.42 (0.82)	−0.43	0.90	0.86
E9: Eating^a^	3.60 (0.86)	−0.90	1.12	1.14

^a^Anchored item.

Reversely coded items are in *italics*; misfit values are in **bold.**

### Data analysis

Descriptive analyses yielding means, standard deviations (SDs), and frequencies were used to determine the characteristics of the participants. A major assumption of the Rasch model is unidimensionality for each domain [17,18]. Therefore, before applying the Rasch models to the WHOQOL-BREF, a parallel analysis (PA) [21,22] was used to test the unidimensionality of each domain. PA was used to produce simulation results, and these simulation results were compared to the results from our participants. The number of dimensions was decided based on how many extracted factors had an eigenvalue greater than the generated mean eigenvalue and estimated eigenvalue at the 95th percentile. For example, if the Physical domain has 2 extracted factors with eigenvalues greater than the generated mean eigenvalue and estimated eigenvalue at the 95th percentile, the Physical domain is determined to be 2-dimensional.

Several Rasch RSM models were used to test the item fit of each item on its WHOQOL-BREF domain; that is, we separately examined the items properties in each domain. Therefore, the 2 generic items were not examined in this study. However, it is acceptable not to measure the properties of the 2 generic items because they are often treated as a criterion to validate each WHOQOL-BREF domain e.g., [6,12]. Two fit indices (information-weighted fit statistic [infit] mean square [MnSq] and outlier-sensitive fit statistic [outfit] MnSq) were used, and the MnSq range of 0.6 to 1.4 suggests an acceptable fit [23]. Specifically, an item with a fit statistic > 1.4 means that the item may not contribute to the same underlying construct as do the other items in the same scale. An item with a fit statistic < 0.6 means that the item may be redundant in the same scale [24]. The Rasch rating-scale model can report standardized item difficulties with a mean of 0 and an SD of 1 log-odd unit (i.e., *logit*). A higher *logit* represents a more difficult item.

Item and person separation reliability, separation index, category functioning, local dependency, and person fit statistics have also been assessed using Rasch RSM models. Person separation reliability was measured by the reproducibility of person ordering on respondent abilities when they answer another set of items measuring the same concept, while item separation reliability was evaluated by the reproducibility of hierarchical item difficulty when the same items were answered by another set of respondents with comparable ability. We applied separation indices to examine how well the respondents can be discriminated (person separation index) and by how well the items can be separated (item separation index) using questionnaires [18]. An acceptable value for person and item separation reliability is > 0.7 [24], while that for person and item separation indices is >2, indicating that the measure can separate respondents (person separation index) or items (item separation index) into more than 2 distinct groups [19].

Category functioning means whether successive response categories for each item are located in their expected order; for example, the difficulty of the response “Not at all” should be lower than that of the response “Slightly” on item “How well are you able to concentrate?” In order to examine the category functioning, average measure (the estimates of average ability on a particular category that is chosen by all respondents), step measure (the thresholds and the boundaries between categories), and category fit statistics (the infit and outfit MnSq) were used. Both average and step measures are expected to monotonically increase with categories, while both fit statistics are recommended to be between 0.6 and 1.4 [18,19].

Local dependency, which means that some items are still correlated after the same underlying concept has been taken into account (e.g., the same wordings), was examined using the correlations (*r*) of the Rasch residuals between every two items. If there is no local dependency, the *r* will be 0; however, Wang et al. [25]: p. 5 claimed that “…there is always some degree of local dependence in empirical data.” Therefore, some degree of local dependence (say, *r* ≤ 0.4) would still be acceptable [26].

Person fit was also examined using infit and outfit MnSq, where an infit or an outfit MnSq value > 1.4 indicates a misfit respondent. Using the person fit statistics, the person response validity can be examined for logical hierarchical ordering [27]. Fisher et al. [28] suggested that the person response validity of children’s school task-related measures could be established when < 5% of children is misfit. However, we decided not to adopt such a criterion, and simply reported the percentage of misfit respondents because our sample had a special mental health issue. Finally, items were tested for differential item functioning (DIF) across educational level (junior high vs. senior high), gender (female vs. male), HBV (positive vs. negative), HCV (positive vs. negative), and HIV (positive vs. negative).

Rasch analyses were done using WINSTEPS [26], and other analyses were done using SPSS 16.0 (SPSS Inc., Chicago, IL, USA).

## Results

The mean (SD) age of the participants was 38.07 (7.44) years, and the mean for first use of heroin was at age 26.13 (6.32). Their mean duration of heroin use was 8.05 (5.85) years. Most participants (*n* = 212, 89.8%) were male, and had 9.43 (2.35) years of formal education. In addition, 19.9% were HIV-positive; 16.5% were HBV-positive; and 94.5% were HCV-positive. Moreover, 155 participants had simultaneously used methamphetamine and heroin (Table 2).

**Table 2:** **Demographic characteristics of participants (** ***N*** **= 236)**

**Variables**	***n*** **(%) or Mean (SD)**
*Demographic*	
Age (years)	38.07 (7.44)
Male gender	212 (89.8%)
Living alone	15 (6.4%)^a^
Years of formal education	9.43 (2.35)
Had fixed employment	125 (53.2%)^b^
*Family history of drug abuse*	33 (14.0%)
*Medical history*	
Age at first heroin use	26.13 (6.32)
Duration of heroin use	8.05 (5.85)^c^
Concurrently use methamphetamine	155 (65.7%)
HIV carrier positive	47 (19.9%)
HBV carrier positive	39 (16.5%)
HCV carrier positive	223 (94.5%)

Human immunodeficiency virus, HIV; hepatitis C virus, HCV; hepatitis B virus, HBV.

^a^ *n* = 233 due to missing answers.

^b^ *n* = 235 due to missing answers.

^c^ *n* = 196 due to missing answers.

The PA showed that only the first factor extracted from each domain had a higher observed eigenvalue as compared to the estimated eigenvalue at the 95th percentile. In addition, the second factor extracted from the Physical domain had the same value (1.15) as the mean eigenvalue from repeated sampling, and the second factor extracted from the other three domains had eigenvalues lower than those from repeated samplings (Psychological: 0.84 vs. 1.11; Social: 0.52 vs. 1.04; Environment: 1.06 vs. 1.20) (Table 3). Because no estimated values at 95% were higher than the observed values in all domains, the PA results suggested that each domain was unidimensional.

**Table 3:** **Observed, resampled mean, and estimated eigenvalues of WHOQOL-BREF**

**Eigenvalue #**	**Observed value**	**Resampled mean**	**Estimated value at 95%**
Physical domain			
1	3.41	1.25	1.33
2	1.15	1.15	1.21
3	0.71	1.07	1.11
4	0.60	0.99	1.04
5	0.46	0.92	0.98
6	0.42	0.85	0.91
7	0.24	0.76	0.83
Psychological domain			
1	3.34	1.22	1.31
2	0.84	1.11	1.16
3	0.73	1.03	1.08
4	0.47	0.96	1.00
5	0.34	0.89	0.94
6	0.27	0.80	0.87
Social domain			
1	2.65	1.15	1.23
2	0.52	1.04	1.10
3	0.45	0.95	1.00
4	0.37	0.85	0.91
Environment domain			
1	4.17	1.30	1.38
2	1.06	1.20	1.26
3	0.86	1.13	1.17
4	0.75	1.05	1.09
5	0.64	0.99	1.03
6	0.46	0.93	0.97
7	0.42	0.87	0.91
8	0.34	0.80	0.86
9	0.31	0.72	0.78

Because each domain was found to be unidimensional, further Rasch analyses were appropriate. Four misfit items were found in the WHOQOL-BREF, and all other items demonstrated acceptable goodness-of-fit in regard to both the infit MnSq and the outfit MnSq. Of the four misfit items, 3 were in the Physical domain (Ph1 [Pain and discomfort]: infit MnSq = 1.77, outfit MnSq = 1.89; Ph2 [Medication]: infit MnSq = 1.56, outfit MnSq = 1.66; Ph6 [Activities of daily living]: infit MnSq = 0.47, outfit MnSq = 0.46) and 1 was in the Psychological domain (Ps6 [Negative feelings]: infit MnSq = 1.56, outfit MnSq = 1.63) (Table 1). The reliability values (item reliability = 0.94-0.98, person reliability = 0.79-0.87) of all four domain scores were acceptable, suggested good internal consistency in each domain. The separation indices (item separation = 4.05-7.65, person separation = 2.06-2.56) of all four domain scores were adequate, except for the person separation index in Physical domain , which was close to the criterion (1.93), indicating good discrimination and separation ability in each domain.

Our results showed that the category functioning of the WHOQOL-BREF followed monotonic increases in average and step measures in four domains, and only three fit indices (Infit MnSq of category 5 in Social domain: 1.41, and of category 5 in Environment domain: 1.46; Outfit MnSq of category 3 in Social domain: 0.59) slightly violated the recommended criterion (Table 4). Therefore, the thresholds of the 5 categories followed the expected order. In addition, the absolute correlations of every two item residuals were all ≤ 0.4, except for two values (Ph2 [Medication] and Ph6 [Activities of daily living] in Physical domain; S2 [Sexual activity] and S3 [Social support] in Social domain), and suggested acceptable local dependency (Table 5). Moreover, the person fit statistics demonstrated that about one fifth of our participants were misfit (Table 6).

**Table 4:** **Threshold disordering tests for each domain on WHOQOL-BREF**

**Domain**				
**Threshold**	**Average measure**	**Step measure**	**Infit**	**Outfit**
**Physical**				
1	−1.43	--	1.10	1.17
2	−0.65	−2.96	1.12	1.27
3	−0.03	−0.86	0.87	0.86
4	1.21	0.21	0.87	0.89
5	2.53	3.62	1.04	0.98
**Psychological**				
1	−2.42	--	1.19	1.25
2	−1.29	−2.80	1.01	1.04
3	−0.19	−1.29	0.86	0.83
4	1.32	0.59	0.93	0.92
5	2.87	3.49	1.13	1.07
**Social**				
1	−3.57	--	1.28	1.33
2	−2.34	−4.31	0.96	1.11
3	−0.70	−2.30	0.69	**0.59**
4	2.35	0.54	0.97	0.95
5	4.75	6.07	**1.41**	1.06
**Environment**				
1	−2.56	--	1.02	1.09
2	−1.35	−2.98	0.98	1.00
3	−0.25	−1.43	0.85	0.82
4	1.34	0.29	0.92	0.93
5	2.84	4.12	**1.46**	1.18

Misfit values are in **bold.**

1 = Very poor/Very dissatisfied/Not at all/Never/None at all.

2 = Poor/Dissatisfied/A little/Slightly/Seldom/Slight/Sometimes.

3 = Neither poor nor good/Neither satisfied nor dissatisfied/A moderate amount/Moderately/Quite often/Moderate/Rather frequently.

4 = Good/Satisfied/Very much/Very/Mostly/Very often/Great/Very frequently.

5 = Very good/Very satisfied/An extreme amount/Extremely/Completely/Total/Always.

**Table 5:** **Correlation coefficients of residuals between items for Item dependency tests**

**Domain**		***r***	**Domain**		***r***	**Domain**		***r***	**Domain**		***r***
**Item #**	**Item #**		**Item #**	**Item #**		**Item #**	**Item #**		**Item #**	**Item #**	
**Physical**			**Psychological**			**Environment**			**Environment**		
Ph1	Ph2	0.02	Ps1	Ps2	−0.20	E1	E2	0.12	E4	E5	0.11
	Ph3	−0.31		Ps3	−0.13		E3	−0.26		E6	−0.22
	Ph4	−0.30		Ps4	−0.32		E4	−0.25		E7	−0.24
	Ph5	−0.28		Ps5	−0.26		E5	−0.10		E8	−0.24
	Ph6	−0.40		Ps6	−0.25		E6	−0.15		E9	−0.15
	Ph7	−0.34	Ps2	Ps3	−0.004		E7	−0.06	E5	E6	−0.30
Ph2	Ph3	−0.20		Ps4	−0.23		E8	−0.23		E7	−0.34
	Ph4	−0.24		Ps5	−0.11		E9	−0.14		E8	−0.16
	Ph5	−0.32		Ps6	−0.32	E2	E3	−0.39		E9	−0.15
	Ph6	*−0.43*	Ps3	Ps4	−0.19		E4	−0.13	E6	E7	0.04
	Ph7	−0.23		Ps5	−0.21		E5	−0.13		E8	0.02
Ph3	Ph4	−0.10		Ps6	−0.26		E6	0.17		E9	−0.11
	Ph5	0.08	Ps4	Ps5	0.17		E7	−0.11	E7	E8	0.13
	Ph6	−0.04		Ps6	−0.30		E8	−0.25		E9	−0.15
	Ph7	−0.14	Ps5	Ps6	−0.25		E9	−0.24	E8	E9	−0.10
Ph4	Ph5	−0.20				E3	E4	0.09			
	Ph6	0.09	**Social**				E5	−0.07			
	Ph7	0.06	S1	S2	−0.35		E6	−0.24			
Ph5	Ph6	0.12		S3	−0.31		E7	−0.22			
	Ph7	−0.24		S4	−0.25		E8	−0.06			
Ph6	Ph7	0.23	S2	S3	*−0.43*		E9	0.02			
				S4	−0.30						
			S3	S4	−0.34						

Two |*r*| values > 0.4 are in *italics.*

**Table 6:** **Summaries of person fit**

	**Mean (SD)**	**Range**	***n*** **(%) [infit/outfit > 1.4]**
Physical			
Infit	1.00 (0.73)^a^	0.08-3.73^a^	56 (23.7%)
Outfit	1.02 (0.76)^a^	0.07-3.96^a^	60 (25.4%)
Psychological			
Infit	1.01 (0.91)	0.05-7.37	50 (21.2%)
Outfit	1.00 (0.91)	0.05-7.18	50 (21.2%)
Social^b^			
Infit	0.92 (1.18)	0.03-6.62	42 (17.8%)
Outfit	0.93 (1.24)	0.03-7.65	48 (20.3%)
Environment			
Infit	0.98 (0.79)^c^	0.10-5.60^c^	50 (21.2%)
Outfit	0.98 (0.78)^c^	0.08-5.43^c^	51 (21.6%)

^a^Participants with maximum extreme score were not included (*n* = 3).

^b^Participants with maximum extreme score (*n* = 2) or minimum extreme score (*n* = 1) were not included.

^c^Participants with maximum extreme score were not included (*n* = 1).

No DIF items were detected on WHOQOL-BREF across either the HBV-positive and HBV-negative carriers or the HCV-positive and HCV-negative carriers. Items E9 (Eating) and S2 (Sexual activity) were found to be DIF items for educational level and gender, respectively. For the participants with the same QoL, those with a junior high educational level and below tended to score higher than those with a senior high educational level and above on item E9 (DIF contrast = 0.50), and females were prone to score lower than were males (DIF contrast = −0.86) on item S2. Five items were found to have a DIF between HIV-positive carriers versus HIV-negative carriers. For the participants with the same QoL, HIV-positive carriers tended to score higher on Ps3 (Think; DIF contrast = 0.63) and S2 (Sexual activity; DIF contrast = 0.64), and tended to score lower on Ps6 (Negative feelings; DIF contrast = −0.62), S1 (Personal relationship; DIF = −0.71), and E7 (Health service; DIF contrast = −0.57) than HIV-negative carriers (Table 7).

**Table 7:** **Differential item functioning (DIF) items detected across different groups**

**Group 1**	**Group 2**	**Item #**	**Item description**	**DIF contrast** ^**a**^	**SE**	**t-value**	**p**
Junior high and below	Senior high and above	E9	Eating	0.50	0.23	2.23	0.03
Female	Male	S2	Sexual activity	−0.86	0.42	2.05	0.048
HIV-positive	HIV-negative	Ps3	Think	0.63	0.24	2.64	0.01
HIV-positive	HIV-negative	Ps6	*Negative feelings*	−0.62	0.24	2.62	0.01
HIV-positive	HIV-negative	S1	Personal relationship	−0.71	0.30	2.37	0.02
HIV-positive	HIV-negative	S2	Sexual activity	0.64	0.29	2.18	0.03
HIV-positive	HIV-negative	E7	Health services	−0.57	0.26	2.20	0.03

^a^DIF contrasts were calculated as: *logit* of Group 1 – *logit* of Group 2; a positive value indicates that a patient in Group 1 has a higher item score than a patient who has the same QoL level in Group 2, and a negative value indicates that a patient in Group 1 has a lower item score than a patient who has the same QoL level in Group 2.

HIV: Human immunodeficiency virus.

Reversely coded items are in *italics.*

## Discussion

To the best of our knowledge, this is the first study using several Rasch models to examine the psychometric properties of the WHOQOL-BREF with a substance-addicted sample. Unidimensionality of the Social and Environment domains was evidenced using both PA and Rasch models; however, the Physical and Psychological domains had misfit items. Three items (Ph1, Ph2, and Ps6) were not embedded in their underlying domain, and item Ph6 was redundant. The WHOQOL-BREF was shown to have satisfactory reliability and separation indices (including person and item). No disordering was detected for 5 thresholds in the four domains, and item dependency was acceptable. However, about one-fifth of the subjects were found to be misfit, possibly indicating the unstable nature of heroin users.

The WHOQOL-BREF has been confirmed as an appropriate QoL instrument around the world e.g., [6,13,29]. Our results of the satisfactory reliability (Physical: 0.96 and 0.79; Psychological: 0.98 and 0.94; Social: 0.94 and 0.82; Environment: 0.98 and 0.87) corroborate with the findings of Fu et al. [6], who used CTT methods on a heroin-dependent sample (Physical: 0.79; Psychological: 0.78; Social: 0.76; Environment: 0.87); of Liang et al. [13], who applied a Rasch model to community-dwelling older people (Physical: 0.76; Psychological: 0.77; Social: 0.68; Environment: 0.78); and of Wang et al. [16], who used a Rasch model on a general Taiwanese population (Physical: 0.86; Psychological: 0.84; Social: 0.83; Environment: 0.82). In addition, we extended the satisfactory reliability to the acceptable separation index. That is, the WHOQOL-BREF has enough items and is sensitive enough to distinguish both high and low QoL participants, and our sample is large enough to verify the item difficulty hierarchy [26].

However, we found that some misfit items in the Physical and Psychological domains contradicted previous Rasch model findings [13,16,29]. One possible reason for this is the different populations used between our study and previous studies (substance abusers vs. a general population and community-dwelling elderly people). Substance abusers are often cognitively impaired [30], and thus may have difficulty understanding some items that use indirect wordings (e.g., negatively worded items). Because three misfit items in this study were negatively worded, we tentatively concluded that substance abusers may not have sufficient intact cognitive function to interpret the three items as they were intended to be understood. Negatively worded items have a wording effect that biases the evaluation of the extracting constructs of QoL instruments [31], especially in the case of people without sufficient cognitive ability. Therefore, the underlying constructs, such as the Physical and Psychological domains, may be affected by the negatively worded items [32,33].

In order to strengthen our hypothesis (i.e., negatively worded items have a wording effect on substance abusers), we used another statistical method (confirmatory factor analysis, CFA) to justify the results. Two CFA models (M1: 4-QoL-factor model, and M2: correlated QoL traits and uncorrelated wording methods) were compared, and we hypothesized that M2 outperformed M1 because it includes the wording effect in the model. Our results showed that M2 substantially improved the data-model fit in χ^2^ difference test (∆χ^2^ = 149.81, ∆*df* = 23; *P* < 0.001), expected cross-validation index (ECVI; M1: 3.894, M2: 3.166), and Akaike information criterion (AIC; M1: 895.673, M2:728.120). The CFA results, therefore, somewhat confirmed our hypothesis. However, because no cognitive tests were done in this study, our hypothesis was only supported by indirect evidence (i.e., Rasch and CFA models). Therefore, future researchers may want to verify our hypothesis using direct investigations. For example, cognitive interviews can be conducted to clarify whether substance abusers have insufficient cognitive functioning by which to understand negatively worded items. In addition, DIF analyses among substance abusers and non-abusers on negatively worded items may also justify our hypothesis.

Our results suggested that the WHOQOL-BREF exhibited the expected threshold ordering among the five categories and low item dependence for a sample of heroin users. The major reasons for this may be a combination of following factors: excellent instruction documents originally provided by the WHOQOL team for the establishment of the version for Taiwan, sound leadership and cooperation among psychometricians, clinicians, and statisticians in Taiwan to form a focus group for the development, careful selection of descriptors for each item [34], standard translation procedure (i.e., forward translation, backward translation, and reconciliation), as well as active participation from 17 hospitals/clinics throughout Taiwan [12]. Future studies are warranted for corroboration of our findings in people with other mental illnesses.

A substantial percentage (17.8% to 25.4%) of person misfit was found in our heroin-dependent patients, which is much higher than 5%, as suggested by Fisher et al. [28]. However, such high percentages might be largely explained by following reasons: First, all of the people in our sample had a diagnosis of opioid dependence, and they are frequently associated with mood problems and/or impaired cognition. Second, the less than 5% misfit was suggested for children without any mental health problems in cases where the objective ability to complete school tasks was being measured [28]. Because we assessed subject reported outcomes from patients, which are frequently affected by emotion and cognition [35,36], less than one-quarter of person misfit may indeed be acceptable. However, more studies are needed to corroborate our speculation.

Although the DIF analyses did not detect any item for HBV or HCV infection, we did find 5 DIF items for HIV infection. Namely, WHOQOL-BREF should be measured and interpreted in opioid users with and without HIV infection separately because 5 out of 26 items were DIF items. In addition, we found one DIF item for educational level and another one for gender. The participants with less than a junior high school education seemed to overestimate eating satisfaction, or they seemed to be more easily satisfied than those with higher education and thus to report a higher score on item E9. Furthermore, the reason that females report an underestimated sexual life QoL may be due to their embarrassment or higher expectations.

This study has three main limitations. First, all participants were recruited in the same MMT program in southern Taiwan, which prevents us from generalizing our results to the entire Taiwanese population. However, our results were comparable to those of another study [6] testing psychometric properties of WHOQOL-BREF in substance abusers from northern Taiwan. Therefore, the generalizability issue may not be serious. Second, the participants were recruited from an MMT program; thus, our results may not be applicable to substance abusers who do not seek anti-addiction treatment. Third, all participants in this study used heroin, and only some used other substances (e.g., methamphetamine, ketamine). Therefore, our results may be more representative of the heroin-dependent population and less representative of populations dependent upon other substances.

In conclusion, the WHOQOL-BREF is suitable to use for evaluating the QoL of substance abusers. It can also be used as a treatment outcome measure to evaluate the effect of treatments for substance abusers. However, those with and without HIV infection should be interpreted after stratification, and the three negatively worded items should be used with caution because substance abusers may have cognitive problems that may preclude them from having a full understanding of the meanings. Future research may apply cognitive interviews to determine the cognitive functioning of substance abusers and their interpretation of negatively worded items.

## Abbreviations

CTT: Classical test theory
QoL: Quality of life
WHOQOL-BREF: The brief version of the World Health Organization Quality of Life assessment
DSM-IV: The Diagnostic and Statistical Manual of Mental Disorders, 4th edition
MMT: Methadone maintenance treatment
PA: Parallel analysis
HIV: Human immunodeficiency virus
HBV: Hepatitis B virus
HCV: Hepatitis C virus
